# Bone Tissue Bioengineering for Craniofacial and Dental Applications: Association of Deciduous Dental Pulp Stem Cells to Carbonated Hydroxyapatite

**DOI:** 10.3390/ijms27042005

**Published:** 2026-02-20

**Authors:** Nidia Silva Marinho, Carla Cristina Gomes Pinheiro, Adriana Terezinha Neves Novelino Alves, Patricia de Almeida Mattos, Jean Rodrigues Evangelista, Christian Ferreira Bernardi, José Ricardo Muniz Ferreira, Gutemberg Gomes Alves, Guilherme Frederico Bernardo Lenz e Silva, Thiago Schneider Werner Vianna, Monica Diuana Calasans-Maia, Carlos Fernando Mourão, Daniela Franco Bueno

**Affiliations:** 1 Instituto Sírio-Libanês de Ensino e Pesquisa, Hospital Sírio-Libanês, São Paulo 01308-060, Brazil; nidiamarinho@yahoo.com.br (N.S.M.); carlacgp@gmail.com (C.C.G.P.); 2 Department of Oral Surgery, Faculdade de Odontologia, Universidade Federal Fluminense, Rio de Janeiro 24020-140, Brazil; adrianaterezinha@id.uff.br (A.T.N.N.A.); monicacalasansmaia@gmail.com (M.D.C.-M.); 3 Department of Metallurgical and Materials Engineering, Escola Politécnica da Universidade de São Paulo, São Paulo 05508-900, Brazil; s.patricialmeida@gmail.com (P.d.A.M.); guilhermelenz@usp.br (G.F.B.L.e.S.); 4 Faculdade Israelita de Ciências da Saúde Albert Einstein, Hospital Israelita Albert Einstein, São Paulo 05652-900, Brazil; jean.14rod@gmail.com (J.R.E.); chrisfb074@gmail.com (C.F.B.); thiagocirurgia@gmail.com (T.S.W.V.); 5 Department of Bioengineering, R-Crio Stem Cells, Rua Cumaru 204, Campinas 13098-324, Brazil; josericardo@r-crio.com; 6 Cellular and Molecular Biology Department, Biology Institute, Universidade Federal Fluminense, Niterói 24020-141, Brazil; gutemberg_alves@id.uff.br; 7 Department of Clinical and Translational Research, Tufts University School of Dental Medicine, Boston, MA 02111, USA

**Keywords:** tissue bioengineering, mesenchymal stem cells, mesenchymal stem cells derived from the deciduous tooth, carbonated hydroxyapatite

## Abstract

Autogenous grafts remain the gold standard for repairing extensive maxillofacial bone defects, but their associated morbidity motivates the search for alternative strategies in tissue bioengineering. Deciduous dental pulp stem cells (DDPSCs) represent a promising cell source due to their accessibility, multipotency, and osteogenic potential, while nanostructured carbonated hydroxyapatite (cHA) microspheres exhibit biochemical similarity to bone mineral and favorable bioabsorption. This study investigated the osteogenic response induced by the association of DDPSCs with cHA in a rat calvaria critical-size defect model. DDPSCs were expanded, seeded onto cHA microspheres, and characterized in vitro prior to bilateral implantation in 12 Wistar rats, with each animal receiving cHA + DDPSC on the right defect and acellular cHA on the left. After 60 and 90 days, histological and histomorphometric analyses revealed new bone formation in both groups, predominantly from the defect margins toward the center. At 60 days, no significant difference in newly formed bone was observed between groups (*p* = 0.249). At 90 days, the DDPSC + cHA group demonstrated significantly greater bone formation compared with acellular cHA (median 40.70 vs. 11.10 histomorphometric points; *p* = 0.028) and significant reduction in connective tissue (*p* = 0.028). Complete scaffold resorption was observed in all DDPSC-treated defects at 90 days, whereas residual biomaterial persisted in the cHA group (*p* = 0.015), indicating progressive cHA resorption over time. These findings suggest that combining DDPSCs with cHA enhances bone regeneration and that this synthetic, bioabsorbable scaffold represents a promising strategy for future applications in bone tissue engineering.

## 1. Introduction

The reconstruction of extensive craniofacial bone defects remains a major challenge in Dentistry and Medicine. Although autogenous bone grafts are considered the gold standard due to their osteogenic, osteoconductive, and osteoinductive properties, donor-site morbidity and limited availability have stimulated the search for alternative strategies in bone tissue engineering [1,2,3,4]. Among these, the combination of mesenchymal stem cells (MSCs) with biomaterial scaffolds has emerged as a promising approach.

Among the different MSC sources, the dental pulp of exfoliated deciduous teeth provides an attractive reservoir of multipotent stem cells (deciduous dental pulp stem cells, DDPSCs). These cells exhibit high proliferative capacity, ability to differentiate into osteogenic, adipogenic, and chondrogenic lineages, and can be collected through minimally invasive procedures [5,6,7,8,9]. Previous preclinical and clinical studies have demonstrated the potential of DDPSCs to promote bone regeneration when associated with different scaffold materials [10,11,12,13,14,15,16,17]. Moreover, DDPSCs maintain their proliferative and differentiation capacities after cryopreservation, which supports their biobanking and future clinical use.

In addition to selecting a suitable cell source, the choice of an ideal scaffold is central to bone tissue engineering. Collagen sponges, hydroxyapatite (HA), beta-tricalcium phosphate (β-TCP), and deproteinized bovine bone are among the most widely used materials [18,19]. Although these scaffolds can support bone regeneration, their bioabsorption rates, mechanical properties, and clinical performance vary substantially, and none has proven ideal for repairing extensive defects [2,20]. Deproteinized bovine bone (e.g., Bio-Oss^®^) is frequently used due to its structural similarity to mineralized tissue [21]. However, its slow resorption, high-temperature processing, cost, and ethical or religious concerns may limit its use [22].

Nanostructured carbonated hydroxyapatite (cHA) has emerged as a promising synthetic alternative. By incorporating carbonate ions and reducing crystallinity, cHA more closely mimics the mineral phase of native bone and exhibits improved biodegradability compared with stoichiometric HA and xenografts [23,24,25]. Clinical and preclinical studies have shown that cHA contributes to bone formation and undergoes controlled reabsorption in periodontal and maxillofacial applications [26,27,28].

The biological rationale for combining DDPSCs with cHA is supported by the intrinsic osteoconductivity of calcium phosphate scaffolds and the osteogenic, trophic, and immunomodulatory properties of DDPSCs. These cells may enhance bone healing not only through differentiation into osteoblasts but also through the secretion of cytokines, growth factors, and extracellular vesicles that modulate angiogenesis, extracellular matrix deposition, and microenvironment remodeling [6,11,29]. The association to a scaffold with a degradation rate proportional to bone regeneration is particularly important, as materials that degrade too rapidly compromise defect stability, whereas excessively persistent materials hinder bone replacement [28,30,31,32]. Unlike conventional sintered hydroxyapatite or xenogeneic bone substitutes, the nanostructured carbonated hydroxyapatite (cHA) microspheres used in this study are synthesized at physiological temperature and maintain low crystallinity and carbonate substitution, features that more closely mimic native bone mineral and may favor bioresorption and biological performance.

In this context, this study tested the association of human deciduous dental pulp stem cells with nanostructured, non-sintered carbonated hydroxyapatite microspheres testing the hypothesis that this material is capable of modulating bone regeneration dynamics, leading to increased bone formation and greater scaffold resorption when compared with conventional hydroxyapatite, as reported in the literature.

Therefore, the aim of this study was to evaluate the regenerative potential of human DDPSCs associated with nanostructured, non-sintered carbonated hydroxyapatite microspheres in a bilateral rat calvarial defect model. We hypothesized that the association of DDPSCs with biomimetic cHA would enhance new bone formation and accelerate scaffold resorption compared with acellular cHA.

## 2. Results

### 2.1. SEM Analysis of DDPSC Adherence to cHA

**Figure 1:**
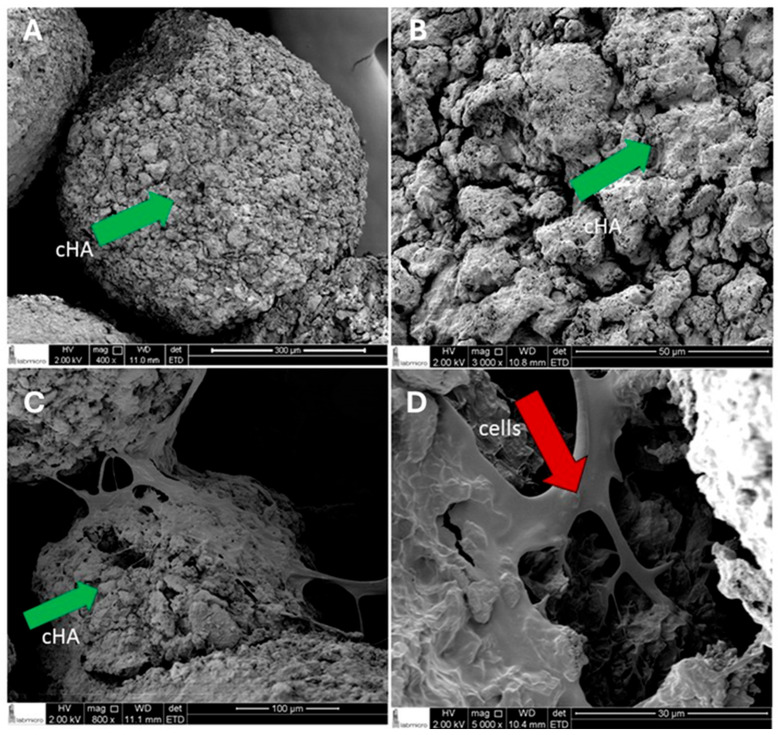
Representative SEM sections (SEM; Inspect F50^®^; FEI Company), (**A**,**B**) showing the porous surfaces of the spheres in samples of the acellular biomaterial: A—400× magnification; (**B**)—Magnification of 3000×. (**C**,**D**) representative SEM sections samples of material made with tissue bioengineering showing the adhesion of deciduous dental pulp stem cells (DDPSCs) to surfaces, with their extensions in the nanostructured carbonated hydroxyapatite (cHA) spheres. (**C**)—Magnification of 800×; (**D**)—Magnification of 5000×. The green arrows indicate the surfaces of the cHA spheres; the red arrow indicates the cell with fibroblastic morphology.

Scanning electron microscopy (SEM) analysis was performed using 3 scaffolds per group and multiple regions of each scaffold were imaged to ensure representative visualization of surface morphology and cell attachment. The analysis was descriptive and qualitative in nature. Nanostructured cHA microspheres exhibited rough and porous surfaces of heterogeneous dimensions (Figure 1A,B). In the samples containing DDPSCs, numerous cells were observed adhered to the surface of the microspheres, forming monolayers with elongated, fibroblast-like morphology (Figure 1C,D). Cell extensions such as filopodia and lamellipodia were visible, demonstrating intimate contact with the cHA surface and intercellular connections. The close arrangement of the cells made individual contours difficult to distinguish, indicating dense cellular organization across the scaffold.

Since the primary focus of the present study is to evaluate the association of dental pulp stem cells (DDPSCs) with cHA, it is important to know that the comprehensive physicochemical characterization of carbonated hydroxyapatite (cHA) synthesized at physiological temperatures has been previously reported by Resende et al. (2019) and Martinez-Zelaya (2019) and the parameters such as crystallinity, phase composition, morphology, Ca/P ratio, specific surface area, pore size, pore volume, and other relevant physicochemical properties are detailed in these earlier publications [26,27]. Therefore, in the present study, we employed the same protocol previously described for cHA synthesis and demonstrated the adhesion of DDPSCs to the cHA scaffold.

### 2.2. Immunophenotyping and Multilineage Differentiation of DDPSCs

**Figure 2:**
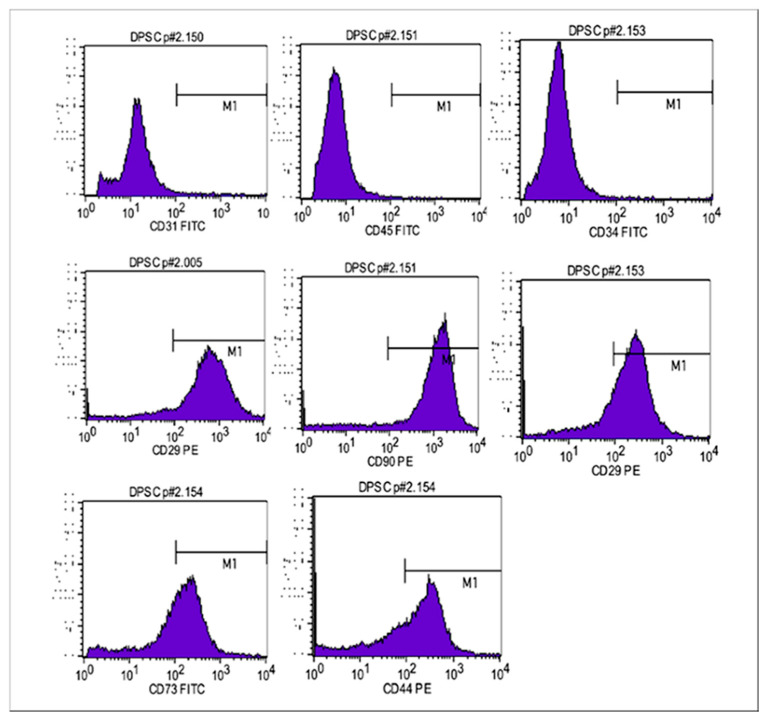
Flow cytometric immunophenotyping of deciduous dental pulp stem cells (DDPSCs). Representative histograms show isotype controls (black lines) and specific antibody staining (purple lines) for mesenchymal, endothelial, and hematopoietic markers.

Flow cytometry confirmed the mesenchymal phenotype of the CTM-PDD population used for the in vivo assays (Figure 2). The cells exhibited high expression of canonical MSC-positive markers, including CD73 (94.31%), CD90 (97.33%), CD105 (96.39%), and CD44 (79.98%), whereas endothelial and hematopoietic markers such as CD31 (1.35%) and CD34 (0.91%) were detected at minimal levels. Overall, the immunophenotypic profile fulfilled the updated ISCT criteria for mesenchymal stromal cells.

**Figure 3:**
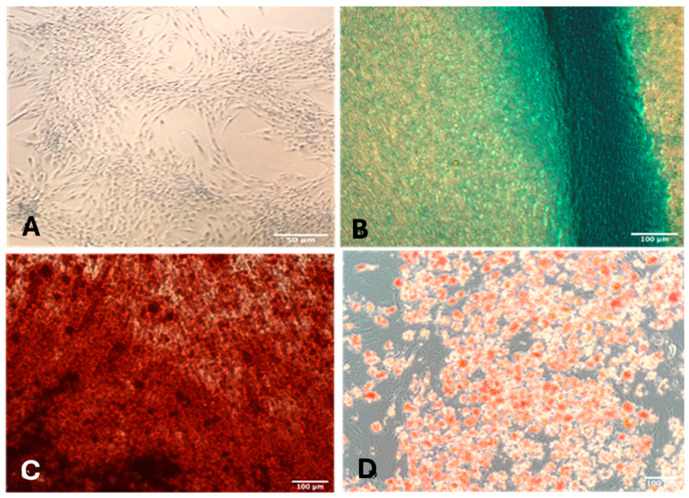
Cell differentiation in mesodermal lines from DDPSCs: (**A**)—negative differentiation control, in 5× magnification with a 50 μm scale; (**B**)—DDPSCs and chondrogenic differentiation after 3 weeks of induction (Alcian Blue), in 10× magnification and 100 μm scale showing extracellular matrix of mucopolysaccharides; (**C**)—adipogenic differentiation with adipocyte staining with red oil and the presence of fat vesicles in 10× magnification and 100 μm scale; (**D**)—DDPSCs and osteogenic differentiation (Alizarin Red), in 10× magnification and 100 μm scale, showing the calcium nodules.

In vitro differentiation assays confirmed the multilineage potential of the DDPSCs (Figure 3). The negative control (Figure 3A) maintained spindle-shaped, fibroblast-like morphology and showed no evidence of lineage-specific matrix deposition. After 3 weeks of chondrogenic induction, cells exhibited Alcian Blue–positive extracellular matrix rich in mucopolysaccharides (Figure 3B). Adipogenic differentiation resulted in the formation of Oil Red O–stained lipid vesicles (Figure 3C), while osteogenic induction produced Alizarin Red–positive calcium nodules (Figure 3D). Together, these findings confirm that the isolated cells met the expected phenotypic and functional criteria for mesenchymal stem cells.

### 2.3. Histological Evaluation of Bone Regeneration

**Figure 4:**
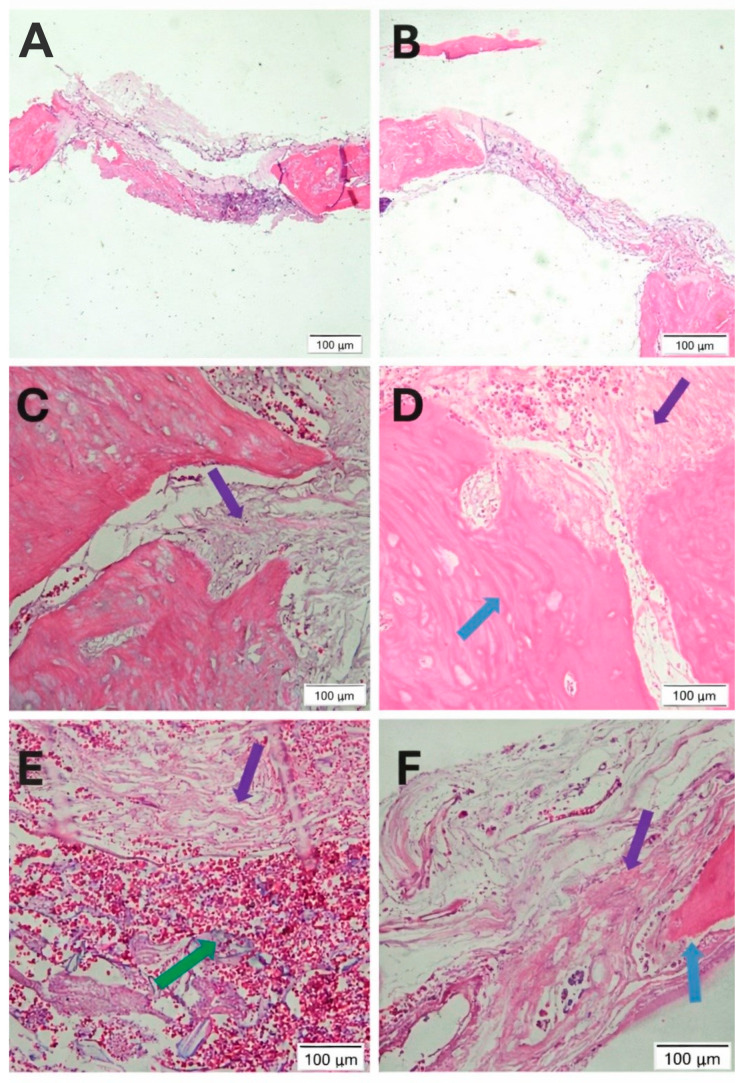
Photomicrographs of experimental groups (acellular cHA control group and cHA + DDPSC tested group) at 60 and 90 days after surgery. (**A**) (acellular cHA) and (**B**) (cHA + DDPSC) 60-days: showing the non-formation of bone and the presence of a large region of fibrocellular tissue; (**C**) (acellular cHA) and (**D**) (cHA + DDPSC) 60-days: with new bone formation (blue arrow) despite the extensive area of connective tissue (purple arrow); (**E**) (acellular cHA) and (**F**) (cHA + DDPSC) 90-days: shows the presence of biomaterial particles (green arrow), new bone formation and connective tissue (blue and purple arrows, respectively). Hematoxylin and Eosin staining. Bar (**A**,**B**): 500 µm; (**C**,**D**): 50 µm. Magnification (**E**,**F**): ×40.

A total of 24 defects (n = 6 per group per time point) were analyzed histologically. After 60 days, both groups exhibited newly formed bone restricted mainly to the borders of the defects, while the central region remained predominantly filled with fibrocellular connective tissue (Figure 4A,B). Fragmented particles of the biomaterial and connective tissue were observed in both groups (Figure 4C,D).

At 90 days, only small remnants of the biomaterial were still present within the defects (Figure 4E,F). The central region predominantly exhibited fibrocellular connective tissue. Areas suggestive of new bone formation were observed mainly at the periphery of the defect and in close proximity to the remaining biomaterial particles (Figure 4E,F). These areas appeared more evident in the DDPSC + cHA group than in the acellular scaffold group. Overall, the histological analysis demonstrated progressive bone formation extending from the margins toward the center of the defect, accompanied by gradual fragmentation and reduction in biomaterial particles and a greater amount of newly formed bone was observed, which was confirmed by histomorphometric analysis.

### 2.4. Histomorphometric Quantification of Newly Formed Bone and Biomaterial

**Figure 5:**
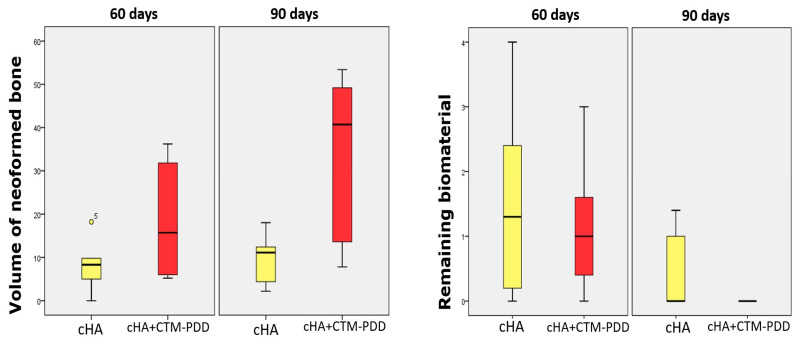
Volume of newly formed bone in the groups cHA (control) and cHA + MSC (experimental), separated between 60 and 90 days of euthanasia; The cHA 90 days versus cHA MSC 90 days; *p* < 0.05. Volume of biomaterial remaining in groups cHA and cHA + DDPSC, separated by period (60 and 90 days); o^5^ Remaining biomaterial cHA + DDPSC 60-d versus remaining biomaterial cHA + DDPSC 90-d; *p* < 0.05.

Statistical comparisons between groups and time points were performed using non-parametric tests, as described in the Materials and Methods. The histomorphometry (Figure 5) revealed no significant difference in newly formed bone between groups at 60 days withing cHA group presented a median of 8.30 points (0.00–18.20), whereas the DDPSC + cHA group showed a median of 15.70 points (5.20–36.20) (*p* = 0.249). Despite the numerical trend favoring the DDPSC-treated defects, variability within this early period limited statistical separation.

At 90 days, however, the regenerative response diverged markedly. The DDPSC + cHA group exhibited a median of 40.70 points of newly formed bone (7.80–53.40), significantly higher than the cHA group (median 11.10; 2.20–18.00) (*p* = 0.028). Connective tissue content also reflected this progression, decreasing significantly in the DDPSC + cHA group relative to cHA alone (*p* = 0.028).

Residual biomaterial measurements demonstrated progressive scaffold resorption in both groups, with a more pronounced reduction in the DDPSC + cHA group. At 60 days, residual cHA accounted for medians of 1.30 points in the control group and 1.00 point in the DDPSC + cHA group. By 90 days, all DDPSC-treated samples revealed absence of detectable residual biomaterial (median = 0.00 points), whereas the cHA group retained small remnants (median 0.00; maximum 1.40). Temporal comparison confirmed that biomaterial loss was significant only in the DDPSC + cHA group (*p* = 0.015).

## 3. Discussion

Bone tissue engineering seeks to combine stem cells with biocompatible scaffolds to overcome the limitations of autogenous grafts, and deciduous dental pulp stem cells have emerged as a promising cell source within this context [7]. In this study, the association of DDPSCs with nanostructured cHA microspheres produced a more favorable regenerative response than cHA alone. To our knowledge, this is the first study to combine human DDPSCs with non-sintered, nanostructured carbonated hydroxyapatite microspheres synthesized at body temperature, addressing a critical gap in the literature and providing a fully synthetic, xeno-free alternative to current clinical standards. The enhanced regenerative response observed in the DDPSC + cHA group at the late healing stage suggests a synergistic interaction between the cellular component and the biomimetic scaffold.

The regenerative behavior observed in the DDPSC-treated defects in this work is consistent with previous studies showing that deciduous pulp stem cells possess robust osteogenic potential in vivo and can effectively contribute to the repair of craniofacial defects when combined with suitable scaffolds [10,11,12,13,14,15,16,17]. Moreover, the maintenance of their proliferative and differentiation capacities after cryopreservation, as previously reported [33,34], further reinforces the translational potential of these cells. In this context, the improved bone formation observed in the DDPSC + cHA group at the late healing stage aligns with the growing body of evidence supporting the capacity of DDPSCs to modulate the regenerative microenvironment and favor bone tissue repair [35,36,37].

Overall, the histological findings demonstrate that while carbonated hydroxyapatite alone provides an osteoconductive scaffold, its association with dental pulp–derived mesenchymal stem cells is associated with histological features suggestive of a more favorable regenerative microenvironment, including improved tissue organization and enhanced new bone formation. Although angiogenesis was not directly assessed, the observed tissue characteristics are consistent with processes that support vascularization and bone regeneration. These findings support the concept that the combined cHA + DDPSC approach may improve both the quality and progression of bone regeneration, reinforcing its translational potential as an Advanced Therapy Medicinal Product.

Beyond osteogenesis, it is important to emphasize that DDPSCs exert potent angiogenic effects, primarily through the secretion of vascular endothelial growth factor (VEGF). VEGF plays a critical role in coupling angiogenesis and osteogenesis by promoting endothelial cell migration, neovascularization, and the delivery of oxygen and nutrients to regenerating tissues [38]. This angiogenic support is essential for sustained bone formation and tissue integration, particularly in critical-sized defects. In addition, mesenchymal stem cells actively modulate the local immune microenvironment through the secretion of anti-inflammatory cytokines and trophic growth factors, thereby establishing a pro-regenerative niche that supports tissue repair and coordinated remodeling [39].

Although molecular markers were not directly assessed, the literature consistently demonstrates that DDPSC osteogenic differentiation involves activation of transcription factors such as RUNX2 and downstream markers including ALP, OPN, and OCN. This established framework provides biological plausibility for the histological findings observed in the present study. Importantly, the coordinated expression of these markers reflects progressive osteogenic maturation and has been consistently reported in mesenchymal stem cell–based bone regeneration models [40]. Although these molecular markers were not directly evaluated in the present study, this well-established molecular framework provides a biological basis to interpret the histological features observed, which are consistent with osteogenic activity in defects treated with the association of DDPSCs and carbonated hydroxyapatite (cHA).

The performance of the nanostructured cHA scaffold observed in this study is also in accordance with previous evidence demonstrating that calcium phosphate biomaterials with lower crystallinity and carbonate substitution exhibit greater bioabsorption and more favorable biological interactions than sintered or xenogeneic hydroxyapatite [23,24,25,26,27]. Unlike deproteinized bovine bone, which undergoes minimal degradation due to its high-temperature processing and dense crystalline structure [21], non-sintered cHA synthesized at physiological temperature tends to dissolve gradually while maintaining osteoconductivity. The progressive reduction in cHA remnants observed in this study, particularly in DDPSC-treated defects, is consistent with prior reports indicating that cHA supports bone formation while undergoing controlled resorption in oral and maxillofacial applications. This behavior is desirable in regenerative therapy, as it reduces long-term scaffold persistence and allows for more complete replacement by newly formed bone.

The enhanced bone formation observed in the DDPSC + cHA group may be related to the complementary biological roles of mesenchymal stem cells and calcium phosphate scaffolds. DDPSCs are known to exert paracrine effects that modulate angiogenesis, extracellular matrix deposition, and osteogenic differentiation through the release of cytokines, growth factors, and extracellular vesicles, thereby influencing the regenerative microenvironment even before differentiating into osteoblast-like cells [41,42]. In parallel, nanostructured cHA provides a favorable osteoconductive surface that supports cell adhesion, as evidenced by the SEM findings. Due to its low crystallinity and carbonate substitution, it is expected to gradually release calcium and phosphate ions during resorption, a mechanism well documented in biomimetic apatites that stimulates osteoblastic activity and mineral deposition [43,44]. A critical requirement in scaffold design is achieving a degradation profile that neither compromises early mechanical stability through overly rapid resorption nor hinders complete bone replacement through excessive persistence. The present results show that non-sintered nanostructured cHA, particularly when combined with DDPSCs, exhibiting progressive and controlled biodegradation that paralleled the increase in bone formation, reaching near-complete resorption by 90 days without evidence of defect collapse or loss of three-dimensional architecture. The combination of these cellular and material-driven mechanisms likely accounts for the more advanced osteogenesis and accelerated scaffold resorption observed in the DDPSC-treated defects at the later healing stage.

The spatial pattern of bone formation observed in both groups—initially concentrated at the defect margins and progressing toward the center—is characteristic of the rodent calvarial model and reflects the osteogenic activity of the native bone edges and the underlying dura mater. The persistence of fibrocellular connective tissue in the central region at both time points is consistent with the limited intrinsic vascularization of this area and has been reported in other studies using similar defect sizes and biomaterials [30,31,32]. Although neither treatment resulted in complete bridging of the defect, the more extensive bone deposition and the reduced amount of residual scaffold in the DDPSC + cHA group at 90 days indicate a more advanced stage of healing. These findings suggest that the combined approach may better support the gradual replacement of biomaterial by newly formed tissue, despite the inherent limitations of this experimental model in achieving full defect closure within the evaluated timeframe. The choice of 60 and 90 days allowed the capture of both the early osteoconductive phase, when biomaterial remnants were still clearly present, and the late remodeling phase, during which the beneficial effect of DDPSCs on bone formation and scaffold resorption became statistically significant [30,31,32].

Although the present findings strengthen the evidence supporting the use of DDPSCs associated with cHA in bone regeneration, several limitations must be acknowledged. The rat calvaria defect, while widely accepted, does not reproduce the mechanical and dimensional complexity of load-bearing craniofacial defects, and the intrinsic regenerative capacity of the dura mater may partially influence healing dynamics. Furthermore, only two postoperative periods were evaluated, which limits the understanding of earlier cellular events and of long-term remodeling. The study relied on DDPSCs derived from a single donor to ensure experimental consistency; however, this design does not capture potential inter-donor variability, which should be addressed in future investigations. Additionally, these analyses focused on histological and histomorphometric outcomes; molecular assessments of osteogenic markers or angiogenic factors could provide deeper mechanistic insights but were beyond the scope of this work. Importantly, none of these limitations compromise the main conclusions of the study, as the differences between groups were consistent across all experimental endpoints and aligned with established biological behavior in this model.

Future studies should explore the translational potential of this combined strategy by extending the healing periods and incorporating larger animal models that better reproduce the biomechanical and anatomical characteristics of human craniofacial defects. Complementary analyses of angiogenesis, inflammatory modulation, and the expression of osteogenic markers could further elucidate the mechanisms by which DDPSCs interact with cHA throughout the regenerative process.

Comparative studies involving commercially available scaffolds, such as deproteinized bovine bone or sintered hydroxyapatite, would also help contextualize the performance of non-sintered cHA within current clinical alternatives. In addition, assessing cell behavior under Good Manufacturing Practice conditions and evaluating inter-donor variability will be essential steps toward the future clinical translation of DDPSC-based therapies. Collectively, these directions may provide a more comprehensive understanding of the biological and translational advantages of combining DDPSCs with nanostructured cHA.

## 4. Materials and Methods

### 4.1. Cell Isolation, Culture, and Expansion

A biological sample containing undifferentiated cells isolated from the dental pulp of a deciduous tooth from an individual with a non-syndromic cleft lip and palate was included in this study after obtaining the informed consent signed by the parents, in accordance with the guidelines of Sírio-Libanês Hospital’s Ethics Committee (CEP: 1.197.470; HSL Ethics Committee number: 5461 (2021) and Plataforma Brasil number: 12406113.7.0000.5461—2021). The DDPSCs were isolated, stored, and cryopreserved at the laboratory of Good Manipulation Practices (GMP) at Sírio-Libanês Hospital’s Cell Technology Center, following previously established and validated protocols [9,11,17].

Cells were grown at 37 °C with 5% CO_2_ with a basal medium composed of Dulbecco’s Modified Eagle Medium/Nutrient Mixture F-12 (DMEM; Gibco (Thermo Fisher Scientific, Waltham, MA, EUA), supplemented with 15% fetal bovine serum (FBS; GE HealthCare, Boston, MA, EUA), 100 U/mL Penicillin-Streptomycin (Gibco, EUA) and non-essential amino acids (Gibco, EUA). The cells were expanded using Trypsin (Gibco, EUA).

Cell characterization was performed by flow cytometry using a FACSCalibur instrument (BD Biosciences, San Jose, CA, USA) and analyzed with CellQuest software version 5.1.0 (BD Biosciences, San Jose, CA, USA). For immunophenotyping, adherent cells were harvested at a density of 1 × 10^6^ cells and incubated with monoclonal antibodies against the mesenchymal markers CD29, CD44, CD73, CD90, and CD105, as well as against the endothelial marker CD31 and hematopoietic markers CD34 and CD45. The fluorochrome-conjugated antibodies used included CD31-FITC, CD34-FITC, CD45-FITC, CD44-PE, CD73-PE, CD73-FITC (for validation), CD90-PE, CD105-PE, CD29-PE, and corresponding isotype controls IgG-FITC and IgG-PE (all from BD Biosciences, San Jose, CA, USA). DDPSCs were selected based on the SSC (side scatter) parameter, with positive marking defined as fluorescence intensity above 10^2^ for each fluorochrome, and at least 1 × 10^5^ cells acquired per sample.

Multilineage differentiation was assessed as previously described [9,11] to confirm the functional identity of the DDPSCs. Chondrogenic medium induced the formation of extracellular matrix rich in sulfated mucopolysaccharides, evidenced by Alcian Blue staining. Adipogenic induction resulted in intracellular lipid vacuoles positively stained with Oil Red O. Osteogenic differentiation was verified by the presence of mineralized nodules stained with Alizarin Red. Collectively, these assays confirmed that the isolated cells fulfilled the phenotypic and functional criteria expected for human mesenchymal stem cells.

### 4.2. cHA Scaffolds

The comprehensive physicochemical characterization of carbonated hydroxyapatite (cHA) synthesized at physiological temperatures has been previously reported [26,27]. In those studies, cHA was extensively characterized in terms of crystallinity, phase composition, morphology, Ca/P ratio, surface area, pore size, pore volume, and other relevant physicochemical properties. The same established methodology was used in the present study to produce the biomaterial. It is important to emphasize that this manuscript was designed to build directly upon these prior findings and to focus specifically on the in vivo biological evaluation of the previously characterized cHA material in combination with DDPSCs.

### 4.3. Preparation of the DPSC+cHA Scaffolds

The cHA scaffold used in this work was produced at the Bioceramics Laboratory of CBPF (Rio de Janeiro, Brazil) using a biomimetic precipitation route. An aqueous solution of dibasic ammonium phosphate was added dropwise to a calcium nitrate solution maintained at 37 °C under continuous stirring, with the pH adjusted to a strongly alkaline range to favor the formation of a carbonated, low-crystallinity apatite. The resulting precipitate was dried and sieved to obtain a fine powder, which was then combined with sodium alginate and dripped into a calcium chloride solution to generate microspheres by ionic crosslinking. After washing and controlled drying, microspheres in the 425–600 µm size range were selected and sterilized prior to use. The physicochemical properties of this material, including carbonate substitution, nanostructured morphology, and low crystallinity, were characterized in detail in a previous study [33]. Briefly, the synthesized cHA exhibited approximately 6% carbonate incorporation, nanocrystallite sizes in the 20–60 nm range, and a porous, low-crystallinity surface architecture consistent with biomimetic carbonated apatites.

DPSCs suspended in 100 µL were seeded in pre-moistened scaffolds at a density of 1 × 10^6^ cells/5 mg of cHA microspheres for 24 h. After 24 h, the biomaterials with the cells were packed in 2 mL of osteogenic induction medium StemPro^®^ (Gibco, USA) and kept in a humid atmosphere at 37 °C with 5% CO_2_ for 10 days. A cell-free scaffold was used as control group, being only moistened with the same amount of osteogenic induction medium. A total of 24 scaffolds were prepared under identical conditions, corresponding to one scaffold per defect. For each time point, six scaffolds were used in the control group (cHA) and six in the experimental group (DDPSC + cHA), ensuring standardization and reproducibility of the experimental conditions.

### 4.4. Assessment of Scaffold Microstructure and Cell Adhesion

The microstructure of the cHA microspheres and the adhesion of DDPSCs to the scaffold were examined using scanning electron microscopy. After scaffold preparation, samples containing the tissue-engineered constructs (DDPSC + cHA + osteogenic induction medium) and samples of the acellular biomaterial (cHA + osteogenic induction medium) were fixed in 2.5% glutaraldehyde. The specimens were then dehydrated through graded ethanol, treated with isoamyl acetate, and vacuum dried. Following dehydration, all samples were sputter-coated with gold and examined using a scanning electron microscope (Inspect F50^®^, FEI Company, Hillsboro, OR, USA) at the Department of Metallurgical and Materials Engineering of the University of São Paulo (USP).

### 4.5. In Vivo Experimental Design and Surgical Procedure

All in vivo procedures were approved by the Ethics Committee on the Use of Animals of Sírio-Libanês Hospital (CEUA/HSL n. 2017-04). There were used 12 Wistar rats of both sexes, three months old and weighing ±300 g, obtained from the Institute of Biomedical Sciences of the University of São Paulo and maintained before and after surgery at the Experimental Animals Laboratory of the Teaching and Research Institute of Sírio-Libanês Hospital, with free access to food and water. Six animals allocated to each experimental period (60 and 90 days). Two critical-size defects were created in each animal, one on the right and one on the left parietal bone, resulting in a total of 24 defects. The right-side defects (n = 12) were filled with DDPSCs associated with cHA (experimental group), while the left-side defects (n = 12) received cHA alone (control group). Consequently, each group comprised six defects per time point.

Under aseptic conditions, anesthesia was induced intraperitoneally by trained personnel certified for in vivo animal procedures by the CEUA, using 1 mL of solution per 100 g of body weight. The anesthetic solution consisted of 0.2 mL tramadol (50 mg/mL), 0.6 mL midazolam, 2 mL ketamine hydrochloride (50 mg/mL), 0.5 mL xylazine 2% (Anasedan^®^ São Paulo, Brazil, 20 mg/mL), and 8.5 mL sterile saline (KabiPac^®^, Fresenius Kabi Brasil Ltda., CE, Brazil). After anesthesia, the surgical field was disinfected with 2% chlorhexidine followed by 0.5% alcoholic chlorhexidine (Rioquímica, SP, Brazil). A straight 1.5 cm midline incision was made along the anterior region of the skull, and the skin and periosteum were carefully elevated with a Molt instrument to expose the calvaria and create a full-thickness flap.

**Figure 6:**
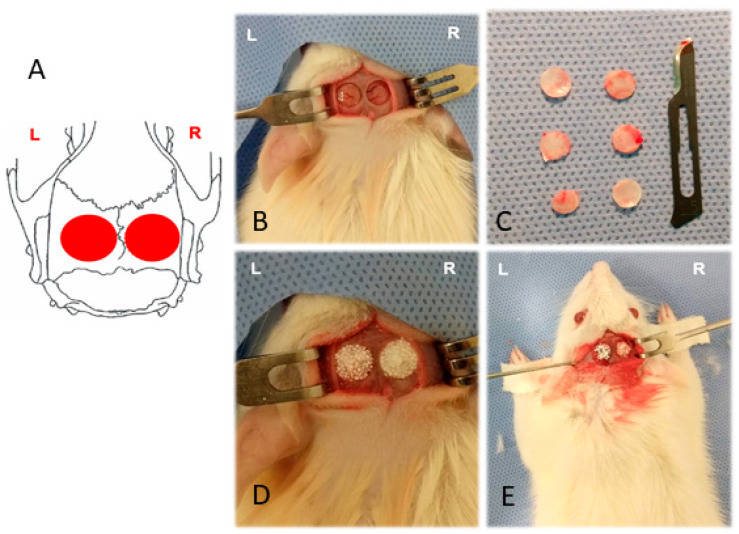
Bilateral manufacture of critical bone defects in the calvaria at the parietal region. (**A**)—Schematic drawing showing the two 5 mm defects in red circles, one on the right side (**R**) of the median sagittal suture and the other on the left side (**L**) of the same size. (**B**)—Defects created showing the exposure of the outermost layer in the dura. (**C**)—Bone fragments removed from calvaria and discarded. (**D**,**E**)—Illustrations of the treatment received by all rats, bone defects filled, right side (**R**) of the calvaria with cHA and MSC-PDT and left side (**L**) with cHA only.

Two standardized circular full-thickness defects measuring 5 mm in diameter and approximately 1 mm in depth were created bilaterally in the parietal bone, one on each side of the sagittal suture. Defects were produced using a trephine bur mounted on a contra-angle handpiece (W&H Dentalwerk, Salzburg, Austria) attached to a low-speed micromotor (Driller^®^, São Paulo, Brazil), set at a rotational speed of 1500 rpm, under continuous irrigation with sterile saline to prevent overheating. Bone fragments were gently removed to preserve the dura mater, as illustrated in Figure 6. All animals received appropriate pre-, intra-, and postoperative care, including analgesic and anti-inflammatory management, and were monitored until recovery. Postoperatively, meloxicam (2 mg/kg, intramuscular) was administered once daily for 3 days, combined with dipyrone (200 mg/kg, intramuscular) every 12 h for the same period.

All 12 rats received both treatments in a bilateral paired design, allowing intra-animal comparison between treatments. This strategy reduces biological variability and increases statistical robustness while adhering to ethical principles for animal research. The defect on the right side was filled with cHA microspheres seeded with DDPSCs (experimental group), whereas the defect on the left side received only cHA microspheres moistened with DMEM-F12 (control group). After implantation, the periosteum and skin were repositioned and sutured with Vicryl^®^ 4-0 (Ethicon, São Paulo, Brazil). Postoperative monitoring was performed daily. Six animals were euthanized after 60 days and six after 90 days using CO_2_ inhalation, followed by removal of the calvaria block for histological and histomorphometric analysis.

### 4.6. Descriptive Histological Analysis and Histomorphometry

Calvarial tissue fragments were fixed in 10% neutral buffered formaldehyde for 24 h and subsequently dissected. After fixation, the specimens were trimmed, labeled, and decalcified. The samples were then embedded in paraffin (Merck, Germany) and sectioned at 5 µm. Hematoxylin–eosin staining (Merck) was performed for morphological evaluation.

For descriptive histology and histomorphometry, the sections were examined under a bright-field microscope (Nikon Eclipse^®^ E400, Tokyo, Japan) equipped with an 80A color temperature correction filter. All analyses were performed by a single examiner blinded to the treatment groups. Five non-overlapping sequential fields were captured from each histological section within the defect region, totaling 120 digital images (5 fields × 24 defects). The percentage of newly formed bone relative to the total defect area was calculated. The percentage of residual biomaterial and connective tissue area relative to the total defect area was also measured to evaluate scaffold bioabsorption and tissue composition.

Quantitative analysis was performed using Image-Pro Plus^®^ 6.0 (Media Cybernetics, Rockville, MD, USA). A grid containing 176 test points was superimposed onto each image to determine the proportion of newly formed bone and residual biomaterial. The volume percentage of newly formed bone was calculated as the ratio of bone volume to total volume within the grid. Statistical analyses were conducted using SPSS^®^ software (version 10.0; Statistics Package for Social Sciences).

### 4.7. Statistical Analysis

The normality of continuous variables was assessed using the Shapiro–Wilk test. Because some variables did not meet the assumptions of normality, nonparametric tests were applied. Paired comparisons between the experimental and control treatments at each postoperative time point (60 and 90 days) were performed using the Wilcoxon signed-rank test. Differences related to euthanasia time were analyzed using the Mann–Whitney U test with Bonferroni correction. A type I error probability of α ≤ 5% and a type II error probability of β ≤ 20% were adopted for all analyses.

## 5. Conclusions

Within the limitations of this experimental model, the association of DDPSCs with nanostructured cHA microspheres enhanced bone formation at 90 days, as demonstrated by quantitative histomorphometric analysis, compared with acellular cHA. This effect was accompanied by a significant reduction in connective tissue content and complete scaffold resorption in the DDPSC-treated defects. These findings indicate that DDPSCs may contribute to a more advanced regenerative response when combined with biomimetic cHA, although further studies are required to clarify the underlying biological mechanisms and potential clinical translation. Future studies will extend healing periods and evaluate this strategy in larger, clinically relevant animal models, incorporating complementary mechanistic analyses (e.g., vascular and inflammatory modulation) to further define the translational potential of this xeno-free, bioabsorbable cell–scaffold platform.

## Data Availability

All data supporting the findings of this study are fully presented within the article. Additional information can be obtained from the corresponding author upon reasonable request.
